# Reed-Sternberg Cells Form by Abscission Failure in the Presence of Functional Aurora B Kinase

**DOI:** 10.1371/journal.pone.0124629

**Published:** 2015-05-01

**Authors:** Ana Xavier de Carvalho, Helder Maiato, André F. Maia, Susana A. Ribeiro, Patrícia Pontes, Wendy Bickmore, William C. Earnshaw, Clara Sambade

**Affiliations:** 1 Instituto de Investigação e Inovação em Saúde, Universidade do Porto, Porto, Portugal; 2 Cell Division Unit, Department of Experimental Biology, Faculdade de Medicina, Universidade do Porto, Alameda Prof. Hernâni Monteiro, Porto, Portugal; 3 The Howard Hughes Medical Institute and the Department of Cellular and Molecular Pharmacology, University of California San Francsicso, San Francisco, United States of America; 4 Instituto de Patologia e Imunologia Molecular da Universidade do Porto, IPATIMUP, Porto, Portugal; 5 MRC Human Genetics Unit, Institute of Genetics and Molecular Medicine, University of Edinburgh, Edinburgh, United Kingdom; 6 Wellcome Trust Centre for Cell Biology, University of Edinburgh, King's Buildings, Mayfield Road, Edinburgh, United Kingdom; University of Nebraska - Lincoln, UNITED STATES

## Abstract

Large multinucleated Reed-Sternberg cells (RS) and large mononucleated Hodgkin cells (H) are traditionally considered to be the neoplastic population in classical Hodgkin lymphoma, (cHL) and postulated to promote the disease. However, the contribution of these larger cells to the progression of cHL remains debatable. We used established cHL cell lines and cHL cellular fractions composed of small mononucleated cells only or enriched in large RS/H cells to investigate RS/H cell origin and to characterize the cells which they derive from. We confirm that the small mononucleated cells give rise to RS/H cells, and we show that the latter proliferate significantly more slowly than the small cells. By using live-cell imaging, we demonstrate that binucleated RS cells are generated by failure of abscission when a few small cells attempt to divide. Finally, our results reveal that the small mononucleated cells are chromosomally unstable, but this is unlikely to be related to a malfunctioning chromosomal passenger protein complex. We propose that the small mononucleated cells, rather than the RS/H cells, are the main drivers of cHL.

## Introduction

Classical Hodgkin lymphoma (cHL) is a neoplasia of B-cell origin, which represents about 10% of all lymphomas showing particular high incidence in teenagers and young adults. The distinctive feature of cHL is the presence of a population of large mononucleated or multinucleated cells, the most typical of which contain two opposing bean-shaped nuclei—Reed-Sternberg cells (RS). The large cells, herein collectively called RS/H cells are considered to be the neoplastic population [1–4] in classical Hodgkin lymphoma and postulated to promote the disease [5–8]. In diseased lymph nodes, RS/H cells exist admixed in an abundant normal population of comparably small B and T lymphocytes, eosinophils, fibroblasts, mast cells and granulocytes. Intriguingly, RS/H cells have been consistently shown to have low proliferative capacity [9–13], and are thought to be derived from crippled germinal center B cells already engaged in early stages of apoptosis [3,4,14]. How the large cell population arises, how it is sustained and how it exerts its neoplastic activity is therefore unclear.

In cell lines derived from the disease and previously established as experimental models for cHL [10,11,15,16], RS/H cells co-exist with a population of smaller, mononucleated cells. Because these smaller cells are mononucleated, they are usually referred to as small Hodgkin cells [9,12]. Studies in the L1236 cell line showed that isolated single small mononucleated cells propagate in culture and can give rise to RS and large H cells, whereas isolated large cells are unable to propagate [12].

The RS cell multinucleation phenotype could be explained either by cell fusion or failure of cytokinesis during exit from mitosis. Studies with cHL patient samples and cHL cell lines, indicated that RS cells are unlikely to form by cell fusion [9,10,17,18]. More recently, time-lapse microscopy of cHL cell lines reported that approximately 83% of RS cells in culture originate from two small sister cells that failed the last stages of cytokinesis [11].

Here, we used cHL cell lines and cellular fractions composed solely of small mononucleated cells or enriched in large RS/H cells to investigate RS/H cell origin. We show that the small mononucleated cells give rise to RS/H cells and that the small cells quickly outgrow the large cells in a population initially enriched in the latter. Our data indicate that binucleated RS cells are generated by failure of abscission when few small cells attempt to divide. Furthermore, our results reveal that the small mononucleated cells are chromosomally unstable, while having a functional chromosomal passenger protein complex.

## Results and Discussion

### Small mononucleated cells quickly outgrow large RS/H cells in culture

For our studies, we used HDLM2, KMH2, L428 and L1236 cHL cell lines, which all showed a morphologic spectrum of small mononucleated cells and large RS/H cells, the latter representing 10–15% of the total population. In order to separate differently-sized cell fractions while preserving cell viability and integrity, we fractionated HDLM2 cells by centrifugal elutriation. Fractions of small mononucleated cells (>98% pure) and fractions enriched in large cells (40% RS/H cells, 60% small cells) were collected and followed in culture during twelve days. RS/H cells started to appear in the cultured small cell fraction by day two after elutriation and their concentration progressively increased. In contrast, the concentration of RS/H cells in the fraction enriched in large cells decreased from ~40% to stabilize at 14%. Twelve days after elutriation, May-Grünwald/Giemsa staining of either fraction was indistinguishable from that of the original HDLM2 cell population, with a concentration of RS/H cells of 11–14% (S1 Fig, panels A, B). These results show that 1) small mononucleated cells give rise to both small mononucleated cells and large RS/H cells, and 2) small mononucleated cells quickly outgrow the large cells in culture.

### Binucleated RS cells are generated by failure of abscission

To investigate how RS/H cells are generated, we imaged the cHL cell lines KMH2, HDLM2 and L1236 by time-lapse microscopy. In all cases, we observed cells undergoing cytokinesis with the cytokinetic furrow ingressing and subsequently regressing. These observations were a strong indication that the small cells that give rise to binucleated RS cells have problems in completing cytokinesis after undergoing mitosis.

**Fig 1:**
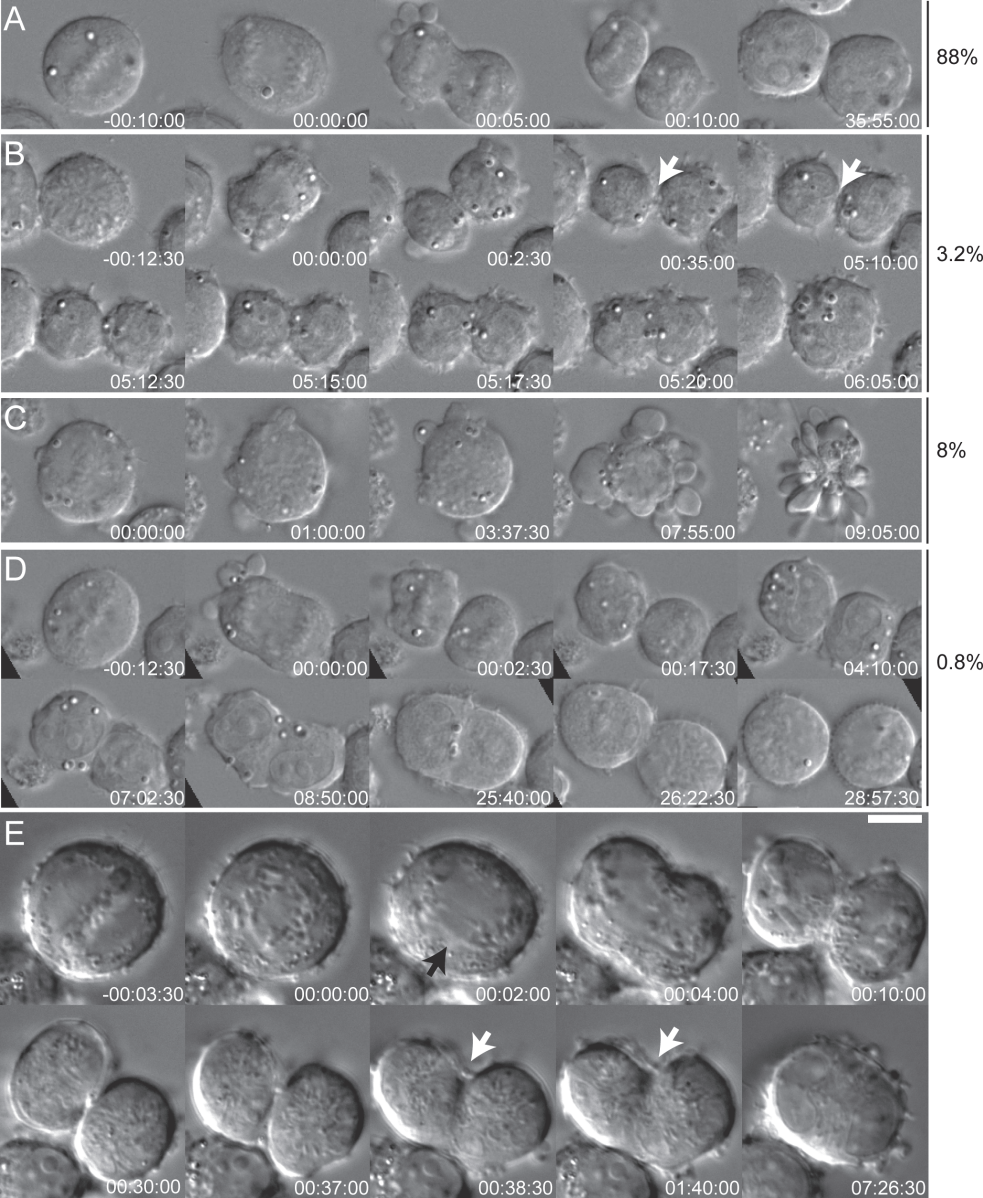
Binucleated RS cells form by failure of abscission. Time lapse video imaging of KMH2 cells undergoing cell division. Shown are selected DIC still images. Time is in hours:minutes:seconds. Time zero is anaphase in A,B,D,E and timepoint after nuclear envelope breakdown in C. **A.** 88% of the cells divide successfully giving rise to two daughter cells. **B.** 3.2% of the cells seem to complete cell division but maintain an intercellular bridge connecting the two cells (white arrow) that later broadens up leading to the formation of binucleated cells. **C.** 8% of cells undergo apoptotic death during mitosis. **D.** < 1% of sister cells appear to fuse but actually remain separate, as the cells round up separately for the following mitosis. **E.** Small mononucleated cell undergoing cell division and failing cytokinesis after completing furrow ingression. The two daughter cells are still connected by the midbody (white arrows), when the furrow regresses. Black arrow points at chromosomal bridges during anaphase. Size bar is 10 μm.

Similar abnormalities were observed when we imaged HDLM2 and KMH2 cells with higher temporal and spatial resolution using DIC microscopy. Quantification of KMH2 cells revealed that of 250 small mononucleated cells undergoing mitosis, 88% completed ingression of the cytokinetic furrow and remained as two separate cells until the end of the recording (Fig 1A and S1 Movie), and 8% died by apoptosis (6.4% after nuclear envelope breakdown, Fig 1C and S3 Movie and 1.6% after completing cleavage furrow ingression). Generation of binucleated RS cells was observed in 3.2% of cells: small mononucleated cells went through mitosis and completed cytokinetic furrow ingression but remained connected by a thin bridge that ultimately regressed giving rise to a binucleated cell (Fig 1B and S2 Movie). The remaining 0.8% cells appeared to fuse after cell division, but what looked like elongated binucleated cells were in fact two individualized sister cells, which separately rounded up for the following mitosis 31 hours later (average KMH2 cell doubling time) (Fig 1D and S4 Movie). When we further increased the temporal resolution by acquiring images every 30 seconds, we observed that the cleavage furrow regressed while the sister cells were still connected by a thin bridge (Fig 1E and S5 Movie). In agreement with the study from Rengstl et al., 2013, our data indicate that RS cells are generated by cleavage furrow regression prior to completion of abscission [11].

### Chromosomal passenger protein complex is functional in cHL

**Fig 2:**
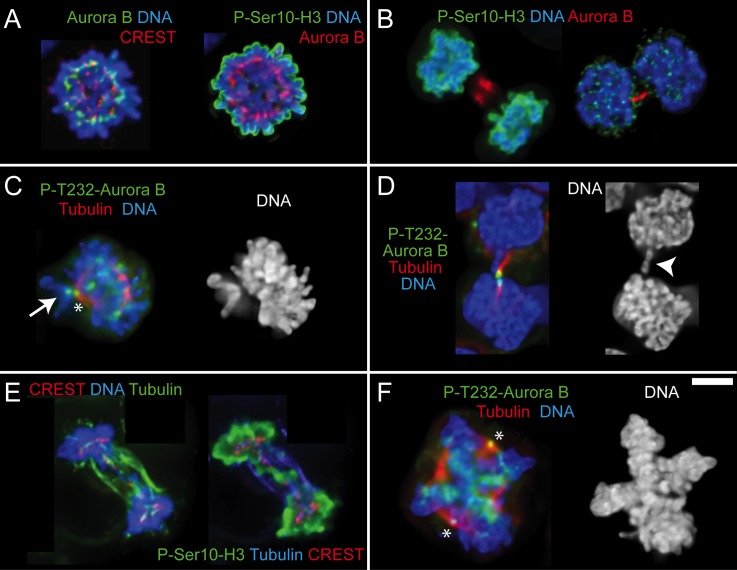
Aurora B activity is normal in cHL small and large cells. A-F. Immunofluorescence images of small mononucleated cells (A-E) and large cell undergoing mitosis (F). A. Cell in prometaphase with Aurora B kinase properly localized at centromeres and phospho-Ser10-histone H3 on chromatin. B. One cell in telophase (left) and one cell in the last stages of cytokinesis (right). Aurora B localizes properly in the central spindle or in the thin intercellular bridge that connects the two sister cells, respectively. As expected, signal for phospho-Ser10-histone H3 is only observed in the cell on the left where nuclear envelope has not reformed yet. C. Cell in prometaphase with most of the chromosomes aligned at the metaphase plate and one mono-oriented chromosome on the spindle pole with the unattached kinetochore showing prominent accumulation of auto-phosphorylated Aurora B (white arrow). Asterisk indicates a spindle pole that is unspecifically labeled by the P-T232-Aurora B antibody. D. Cell in the last stages of cytokinesis with chromatin present in the intercellular bridge (white arrowhead). Auto-phosphorylated Aurora B accumulates on the intercellular bridge revealing that Aurora B has been properly activated at a time when abscission is taking place. E. Cell in telophase with chromatin bridges staining positive for phospho-Ser10-histone H3. F. Large cell with a double metaphase plate showing auto-phosphorylated Aurora B and therefore active kinase on centromeres. Asterisks are the spindle poles. Size bar is 5 μm.

In order to test whether chromosomal instability could be associated with abscission failure, we analyzed KMH2 interphase cells for the presence of micronuclei. We found that 1.2% of the total cell population had micronuclei and of these 99.8% were small mononucleated cells (total number of cells analyzed was 11866). The average number of micronuclei per cell was 1.12 +/- 0.46, with 86.1% having one micronuclei, 10.4% two micronuclei and 3.5% three micronuclei. Analysis of mitotic cells by immunofluorescence in KMH2 cells failed to reveal any obvious enrichment in cytokinesis (5.1% prophases, 62% prometaphases, 11.1% metaphases, 6.4% anaphases and 15.4% cytokinesis, n = 230 mitotic cells). The observation of prometaphases with most chromosomes aligned in the metaphase plate but only a few very close to the spindle poles (Fig 2C) and occasional anaphase/telophase figures containing chromosome bridges (Figs 1E and 2D and 2E) led as to reason that the chromosomal passenger protein complex (CPC)—a master regulator of mitosis and cytokinesis [19]—could be malfunctioning. Indeed, recent work suggested that Aurora B kinase, a member of the CPC, could play a direct role in the development of cHL [20,21]. A malfunctioning CPC might lead to chromosome alignment and segregation defects and could compromise the abscission checkpoint, which delays abscission until chromosome bridges are resolved [22]. The other members of the CPC: Survivin, Borealin and INCENP are essential to activate and localize Aurora B to centromeres during prometaphase-metaphase, as well as to the spindle midzone and midbody during anaphase-telophase and cytokinesis [19].

To investigate whether any CPC member was malfunctioning, we first looked for mutations in Aurora B, Survivin and Borealin. The coding sequences of these genes were sequenced and no mutations were found in mixed cell populations of the HDLM2, KMH2 and L428 cell lines. Second, we looked at Aurora B, Survivin and INCENP localization in the KMH2 cell line mixed population and in the fraction of HDLM2 small mononucleated cells (2 days after elutriation). In all mitotic small or large cells, the localization pattern of Aurora B, Survivin and INCENP was normal (n = 100 for small HDLM2 cells and n = 234 for KMH2 cells, Fig 2 and S1 Fig, panels C, D). We have not looked at Borealin but the normal localization of the other CPC members precludes abnormalities [23]. Immunostaining of mitotic cells with anti-Aurora B phospho-T232, which recognizes the auto-phosphorylation of Aurora B essential for its kinase activity showed that Aurora B is active and functional in these cells (Fig 2C, 2D and 2F) [24]. Properly localized and active Aurora B was observed in all mitotic cells analyzed. Of note is the accumulation of auto-phosphorylated kinase in kinetochores of unaligned chromosomes in prometaphase cells (Fig 2C) and in the thin intercellular bridge in late stages of cytokinesis even when there is chromatin between the two sister cells (Fig 2D). Moreover, all mitotic figures were positive for histone H3 phosphorylated on serine 10, a target of Aurora B, which reinforces the fact that this kinase is active (Fig 2A, 2B and 2E) [25]. We conclude that formation of RS cells is most likely not due to abnormalities in the CPC.

In conclusion, our data demonstrate that RS cells arise from small mononucleated cells that fail abscission in the presence of a functional CPC. Although Aurora B has been previously proposed to be directly involved in the development of cHL, our data reveal that this kinase and the protein complex that it is part of are not malfunctioning in cHL cell lines.

In addition, the observation of micronuclei and chromosome bridges in the small mononucleated cells indicates that these cells are already chromosomally unstable. It becomes therefore essential to determine the fate of small cells with chromosome segregation defects during mitosis or micronuclei during interphase. It also remains to be understood why only a fraction of small mononucleated cells fail abscission and if the cells that fail abscission are able to gain neoplastic features. It will be important to determine whether abscission failure is due to the presence of chromosomal bridges or to the presence of a defective member of the cytokinesis/abscission machinery. A good candidate to study in more detail is KLHDC8B, a protein that localizes to the midbody, is required for successful cytokinesis in HeLa cells and is not expressed or shows decreased expression in some familial cases of cHL [26,27].

Our results show that small mononucleated cells quickly outgrow the large cells in culture, as cell populations enriched for small or large cells both evolve to become identical populations with a constant percentage of ~12.5% of large RS/H cells (which is the average percentage of large cells in cHL cell lines). These data favor the view that small mononucleated cells are crucial drivers of cHL and that RS/H cells are dead-end by-products of an abnormal small mononucleated cell population rather than essential contributors for its neoplastic maintenance. Further investigation will be necessary to understand whether all the small cells are abnormal or whether there is a sub-population of small cells that is responsible for RS/H cell generation as proposed previously [12]. If the cHL cell lines truly behave like the disease, the extrapolation of these data to the in vivo situation represents a major challenge to the conceptual and diagnostic frameworks of cHL.

## Methods

### Cell lines and cell culture

HDLM2, KMH2,L428 and L1236 cells were obtained from DSMZ and grown in suspension cultures in RPMI 1640 medium supplemented with 10% FBS (Gibco-BRL), 100 U/ml penicillin, 100 μg/ml streptomycin and 300 mg/ml L-glutamine. Cultures were grown at 37°C in a 5% CO_2_ atmosphere. All cell lines were maintained at concentrations of < 0.6x10^6^ cells/ml.

### Centrifugal elutriation

Approximately 250x10^6^ HDLM2 cells were spun at 150 x g for 5 minutes at room temperature, resuspended in 10 ml of elutriation buffer (PBS, 1% FBS, 0.1% glucose, 0.3mM EDTA) and loaded into the elutriation chamber. Fractions of small cells were collected at 2650g, flow rate 85 ml/min, and RS/H enriched fractions were collected at 110g, flow rate 120 ml/min. Cell fractions were spun down and immediately resuspended in fresh medium and kept at 37°C.

### Immunofluorescence

Cells were cytospun onto slides or grown on poly-lysine coated coverslips, fixed for 10 minutes with 4% formaldehyde in cytoskeletal buffer (137mM NaCl, 5mM KCl, 1.1mM Na_2_HPO_4_, 2mM MgCl_2_, 2 mM EGTA, 5mM PIPES, 5.5mM glucose) and permeabilized with 0.15% Triton X-100 in cytoskeletal buffer. Slides with cells were immersed in PBS, 1% BSA for blocking for 30 minutes at room temperature. Cells were probed with antibodies against Survivin (Novus-Biologicals), Aurora B (Transduction Laboratories), phospho-T232-Aurora B (Rockland), INCENP (rabbit 1186), α-tubulin (B512, Sigma-Aldrich), CREST and phospho-histone H3(S10) (Cell Signaling). Secondary antibodies were from Molecular Probes, Alexa 488, Alexa 647 and Alexa 568 respectively. Images were acquired on a microscope model IX-70, Olympus controlled by Delta Vision SoftWorx (Applied Precision) using a 60x/1.40 NA objective or on an AxiObserver microscope (Zeiss) with a 100x objective, several Z-sections with an optical slice of 0.22um. Images were deconvolved using SoftWorx or Huygens Professional software (Scientific Volume Imaging) and processed using Fiji software.

For micronuclei quantification, image acquisition was done in an INCell 2000 Analyzer (GE Healthcare) with a 40x/0.95NA objective, over a 1.0 μm thick section. Quantification was done using the Developer Toolbox software (GE Healthcare) and visual data inspection was performed with Spotfire DecisionSite. Mitotic distribution was determined by quantifying fixed cells labeled for α-tubulin, DNA, phospho-histone H3(S10) and phospho-T232-Aurora B. Cells were considered to be in cytokinesis when a cleavage furrow was observed and pT232-Aurora B labeled the spindle midzone or midbody. During telophase, a cleavage furrow is already in place and therefore all telophase figures were included in the cytokinesis category.

### Time-lapse imaging

KMH2, HDLM2 and L1236 cells were seeded at an initial concentration of 0.3x10^6^ cells/mL (96% viability) in a final volume of 2 mL in a 1u-Slide 4 well ibiTreat microscopy chamber (ibidi, Germany). Differential interference contrast (DIC) images were acquired every 2.5 or 0.5 minutes on a Zeiss Axiovert or Nikon TE2000 microscope, using a Plan-Apo 63x/1.40 Oil DIC objective and a CoolSnap HQ camera (Roper) or CoolSnap HQ camera (Photometrics, Tucson, AZ). Total acquisition time was 73 hours. Acquisition software was IP Lab (Scanalytics, Fairfax, VA) or Micro-Manager software and Fiji software was used for image processing.

### RNA extraction and reverse-transcriptase PCR

30x10^6^ cells were homogenised in 10 mL of 3M LiCl, 6M urea and 0.2% SDS and kept at 4°C overnight. Samples were vortexed and spun at 17900 x g for 20 minutes at 4°C. The supernatant was discarded and the pellet was dissolved in 2.5 ml of TES (10 mM Tris-HCl, pH 7.6; 1mM EDTA, pH 8.0; 0.5% SDS). 2.5 mL phenol (pH 4) were added and after agitating vigorously, the samples were centrifuged at 960 x g for 10 minutes. The upper phase was transferred to a new RNAse-free tube and 1/10 volume sodium acetate and 2.2 volumes ethanol were added. Samples were kept at—20°C overnight and were then spun down at 20800 x g for 30 minutes at 4°C. Supernatant was discarded, pellet was air-dried at room temperature and dissolved in TES. RNA quality was verified by agarose gel electrophoresis. cDNA was synthesised using Superscript First-Strand Synthesis (Invitrogen #11904–018), 3 μg of total RNA and oligo(dT). Manufacturer’s instructions were followed. Coding sequences of Aurora B, Survivin and Borealin were amplified from cDNA of HDLM2, KMH2 and L428 cell lines and sequenced. Primers used for amplification were: Aurora B—5' GCCCAGAAGGAGAACTCC3' and 5' CCTTCAATCTGTCGCCT3'; Survivin—5' CCCTTTCTCAAGGACCACC3' and 5' AGCCACTGTTACCAGCAGC3'; Borealin—5'GGGGCTCGAGACATGGCTCCTAGGAAGGGC3' and 5' GGGCGGTACCCCTTTGTGGGTCCGTATGCT3'.

### May-Grünwald/Giemsa staining

Cells were cytospun, air-dried and fixed in methanol for 1 minute. The slides were incubated in Hemacolor II (Merck) for 2 minutes and Hemacolor III (Merck) for 3 minutes at room temperature. Slides were thoroughly washed in water to take the excess of dye off and mounted with Entellan (Merck).

## Supporting Information

S1 Fig RS/H cells derive from small mononucleated cells and the latter proliferate faster than the first in the presence of a functional CPC.A. Homogeneous fraction of small mononucleated cells obtained by elutriation at day zero (A) and after being cultured for 12 days (A'). B. Fraction enriched for large cells obtained by elutriation at day zero (B) and after being cultured for 12 days (B'). C,D. Immunofluorescence images of HDLM2 cells in mitosis showing that Survivin (C), Aurora B, and INCENP (D) localize correctly on centromeres and central spindle during prometaphase and telophase, respectively.
(TIF)

S1 Movie DIC timelapse movie of KMH2 small mononucleated cell dividing successfully and giving rise to two daughter cells. Time is in hours:minutes:seconds. Zero timepoint is anaphase.
(AVI)

S2 Movie DIC timelapse movie of KMH2 small mononucleated cell undergoing mitosis and giving rise to a binucleated RS cell after failing abscission. Time is in hours:minutes:seconds. Zero timepoint is anaphase.
(AVI)

S3 Movie DIC timelapse movie of KMH2 small mononucleated undergoing apoptotic death during mitosis. Time is in hours:minutes:seconds. Zero timepoint is first frame after nuclear envelope breakdown.
(AVI)

S4 Movie DIC timelapse movie of KMH2 small mononucleated cell that completes mitosis and whose daughter cells misleadingly seem to fuse. They do not fuse as they round up in the following mitosis, which proves that the two cells had individualized plasma membranes. Time is in hours:minutes:seconds. Zero timepoint is anaphase.
(AVI)

S5 Movie DIC timelapse movie of HDLM2 small mononucleated cell undergoing cell division and failing abscission after completing cytokinetic furrow ingression. The two daughter cells are still connected by the midbody, when the furrow regresses. Time is in hours:minutes:seconds. Zero timepoint is anaphase.
(AVI)
