# Supplementary figures and images for: Reed-Sternberg Cells Form by Abscission Failure in the Presence of Functional Aurora B Kinase
Source: PLoS One. 2015 May 1;10(5):e0124629. doi: 10.1371/journal.pone.0124629 (PMC4416800; doi:10.1371/journal.pone.0124629)

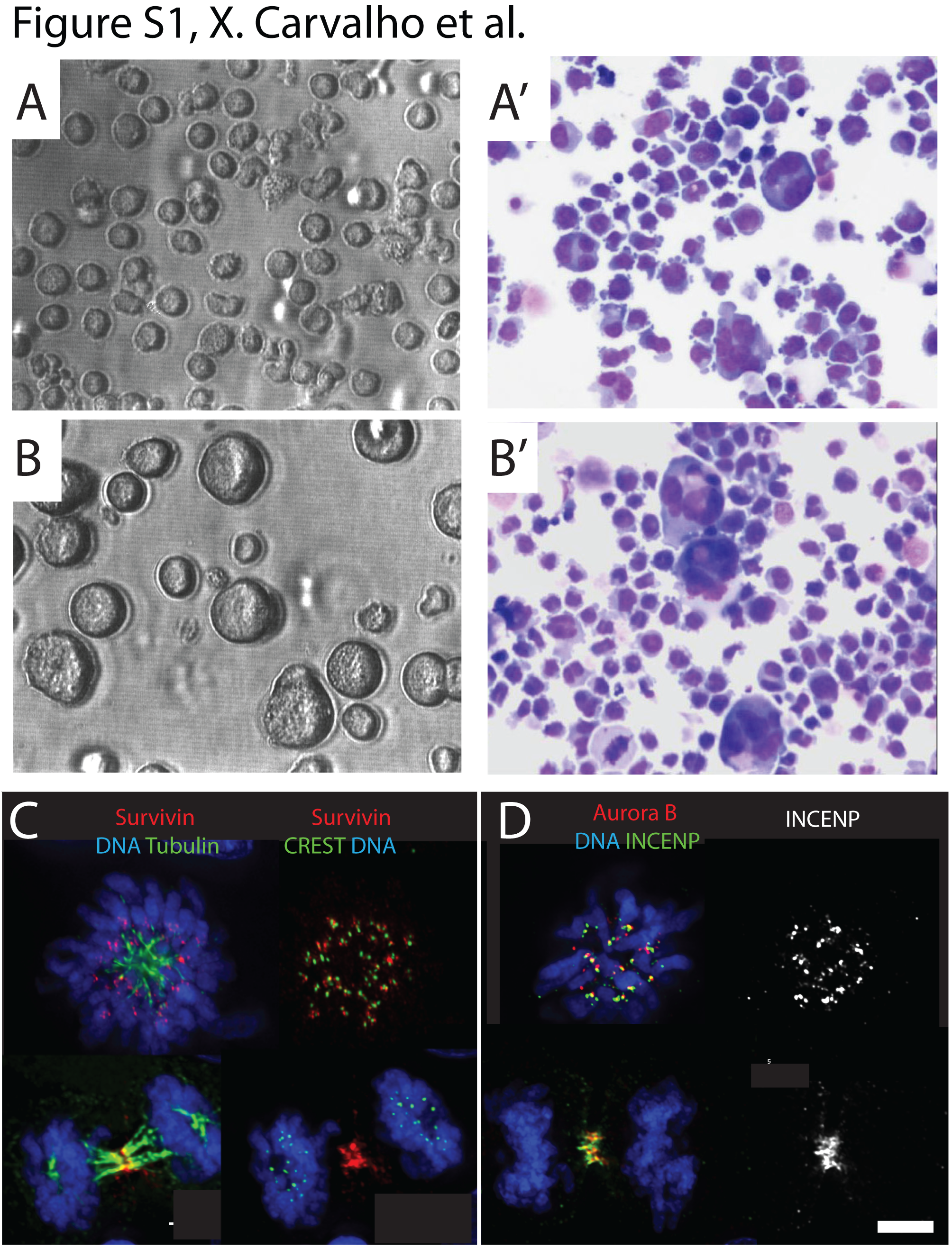

Supplement: S1 Fig — Homogeneous fraction of small mononucleated cells obtained by elutriation at day zero (A) and after being cultured for 12 days (A'). B. Fraction enriched for large cells obtained by elutriation at day zero (B) and after being cultured for 12 days (B'). C,D. Immunofluorescence images of HDLM2 cells in mitosis showing that Survivin (C), Aurora B, and INCENP (D) localize correctly on centromeres and central spindle during prometaphase and telophase, respectively. (TIF) [file pone.0124629.s001.tif]
